# Evaluating soybean yield responses to future climate change and irrigation regimes: a DSSAT multi-model assessment

**DOI:** 10.3389/fpls.2026.1811299

**Published:** 2026-03-27

**Authors:** Alper Baydar, Yeşim Bozkurt Çolak, Mete Özfidaner, Hüdaverdi Gürkan, Engin Gönen

**Affiliations:** 1 Department of Biosystems Engineering, Faculty of Agriculture, Siirt University, Siirt, Türkiye; 2 Department of Biosystems Engineering, Faculty of Agriculture, Malatya Turgut Özal University, Malatya, Türkiye; 3 Alata Horticultural Research Institute, Ministry of Agriculture and Forestry, Mersin, Türkiye; 4 Turkish State Meteorological Service, Ankara, Türkiye; 5 Oil Seeds Research Institute, Ministry of Agriculture and Forestry, Osmaniye, Türkiye

**Keywords:** climate change, DSSAT, global climate model, irrigation, soybean yield, crop simulation model

## Abstract

**Introduction:**

Climate change is expected to intensify temperature and precipitation variability in Mediterranean regions, creating uncertainty for soybean production.

**Methods:**

In this study, the DSSAT-CROPGRO-Soybean model was run with three global climate models under two emission pathways (RCP 4.5 and RCP 8.5) across three future time periods: near-future (2016–2040), mid-century (2041–2070), and late-century (2071–2098). Bias correction significantly enhanced the reliability of climate inputs by reducing systematic temperature deviations and improving agreement with observed meteorological conditions. The model was calibrated and validated using observed phenology, leaf area index (LAI), biomass, and yield across three irrigation treatments (I_100_, I_70_, and I_50_).

**Results:**

The model showed good correspondence between observed and simulated values. Taylor diagram analysis revealed correlation coefficients generally exceeding 0.95, coefficients of determination (R²) ranging from 0.74 to 0.99, and acceptable RMSD values across treatments. Future projections indicated that yield responses varied across future periods and irrigation conditions. Under late-century conditions, RCP 8.5 produced higher yields than RCP 4.5 by approximately 4–17% under irrigated conditions and 6–20% under rainfed conditions across the considered GCMs.

**Discussion:**

Elevated CO_2_ partly mitigated the effects of warming; however, seasonal soil water availability remained the primary constraint on yield. The results demonstrated that the calibrated DSSAT-CROPGRO-Soybean model provides a reliable basis for predicting the adverse effects of future climatic conditions on soybean production, while multi-GCM climate projections indicated that the magnitude of these effects may vary substantially depending on emission scenario, projection period, and water availability.

## 1 Introduction

Soybean (Glycine max L.) is one of the most important oilseed crops worldwide, with global production reaching approximately 420.8 million tons in the 2024/25 season (USDA, 2025). In Türkiye, soybean production reached 180 thousand tons in 2024 and was estimated at 149 thousand tons in 2025 according to official statistics (TURKSTAT, 2024, 2025). Despite its relatively modest share in global production, Türkiye has one of the highest soybean yields in the world, reaching 4.12 t ha^-^¹ in the 2023/24 season (Agricultural Economics and Policy Development Institute, 2024). Soybean cultivation in Türkiye is concentrated mainly in the Mediterranean region, particularly within the Çukurova Basin, where Mersin ranks as the second largest soybean-producing province, contributing about 11.6% of national production (Özcan, 2024). This spatial concentration indicates that soybean production in Türkiye is closely associated with Mediterranean agro-climatic conditions.

Climate change poses a significant threat to agricultural production through rising temperatures, shifting precipitation patterns, and increasing drought frequency can affect crop growth, phenology, and yield development (Timlin et al., 2024). These pressures are particularly pronounced in the Mediterranean region, which is widely recognized as a climate-change hotspot due to accelerated warming and increasing water scarcity (Lazoglou et al., 2024). Crop simulation models have therefore become valuable tools for assessing future climate impacts and identifying potential adaptation strategies in agricultural systems (Singh et al., 2025). While many Mediterranean studies have focused on major cereals such as wheat and maize, relatively few studies have addressed soybean production under projected climate conditions (Koukouli et al., 2025). Moreover, studies evaluating soybean responses to climate change in Türkiye and the eastern Mediterranean region remain limited, highlighting the need for region-specific assessments that integrate crop simulation models with future climate projections.

Recent climate assessments also indicate that the reliability of future climate projections for crops depends strongly on the skill of the global climate models used. Individual GCMs differ substantially in climate sensitivity, the representation of precipitation and the simulation of temperature trends, which can produce substantially different estimates of agricultural impacts even under the same emission scenario (Kamworapan and Surussavadee, 2019). Downscaled and bias-corrected datasets derived from CMIP5 have shown a substantially improved ability to represent local climate variability, particularly in semi-arid regions where strong warming trends and prolonged drying episodes have been documented (Babaousmail et al., 2022). Changes in temperature, evapotranspiration and rainfall patterns directly influence plant physiological processes, integrating multi-GCM and multi-scenario climate inputs into model-based assessments is essential for capturing the full range of uncertainty in long-term projections.

Process-based crop models such as DSSAT have become essential tools for quantifying how climatic drivers affect phenology, crop growth, and yield outcomes (MacCarthy et al., 2022). Their ability to simulate crop responses through physiological processes enables more realistic assessments of how temperature, moisture and radiation interact to shape seasonal development. Recent DSSAT applications further demonstrate that multi-variable calibration is particularly important in environments where heat and moisture stress occur, because parameter uncertainties can otherwise mask climate-driven yield responses (Li et al., 2024). Ensemble-based climate modelling adds robustness to climate change impact assessments by enabling uncertainty to be assessed across alternative GCM projections and scenario (pathway) assumptions (Falloon et al., 2014).

Building on these advances, many regional-scale studies have demonstrated that the CROPGRO-Soybean module reproduces key developmental stages, including flowering onset, pod initiation and physiological maturity when genetic coefficients are calibrated using observational datasets (Ritchie, 1998; Fernandes et al., 2022). Under field conditions, it may not be possible to completely eliminate factors other than those affecting growth and development, along with their interaction with a given production level, and such an approach is also time-consuming and expensive (Nargund et al., 2024). Given soybean’s sensitivity to reproductive heat stress, photothermal constraints and soil-water interactions, the integration of calibrated DSSAT models with multi-GCM climate projections provides a robust framework for quantifying climate risks and designing adaptive management strategies.

Soybean, as a key C3 legume with high sensitivity to temperature and moisture stress during reproductive development, is particularly vulnerable to future climatic changes. Shortened growth duration, accelerated reproductive phenology, reduced canopy expansion and greater pod and seed abortion have been documented in both semi-arid and temperate soybean systems under warming scenarios (MacCarthy et al., 2022; Li et al., 2024). Studies also indicate that even moderate increases in daytime maximum temperature can reduce effective seed-filling duration, thereby decreasing final grain weight despite adequate water availability. Since these physiological responses depend strongly on local climate patterns, cultivar traits and soil-water interactions, region-specific modelling frameworks are required to accurately characterize climate risks and guide adaptive irrigation and management strategies.

To address these gaps, the DSSAT-CROPGRO-Soybean (Version 4.8.5.0) model was calibrated and validated for the study region under different irrigation regimes by comparing field-observed phenological stages and growth indicators with model outputs, ensuring an accurate representation of local crop performance. Following this procedure, the DSSAT model was applied to assess future soybean development and yield using climate projections from three global climate models (HadGEM2-ES, MPI-ESM-MR and GFDL-ESM2M) across three future periods (2016-2040, 2041–2070 and 2071-2098) under two emission pathways (RCP 4.5 and RCP 8.5). This multi-scenario, multi-GCM framework provides a comprehensive basis for evaluating potential impacts of future climate conditions on soybean production in semi-arid environments while capturing uncertainty arising from inter-model variability.

This study offers a distinct contribution by calibrating and validating the DSSAT-CROPGRO-Soybean model under three irrigation regimes, thereby providing a more robust representation of water use and yield responses under Mediterranean conditions. In addition, the integration of bias-corrected climate projections from three global climate models under RCP 4.5 and RCP 8.5 across multiple future time horizons provides a framework for assessing climate-induced changes in soybean productivity. A further novel aspect of this work is the joint evaluation of warming, CO_2_-induced physiological responses, and irrigation management, which together offer a comprehensive understanding of how these interacting drivers shape future yield trajectories and associated water requirements.

## 2 Materials and methods

### 2.1 Description of the experimental area

The research was conducted at the Tarsus location of the Alata Horticultural Research Institute in Mersin, Türkiye (36°53′ N, 34°57′ E; elevation 12 m). The region is characterized by a semi-arid Mediterranean climate, with long-term meteorological observations indicating an annual mean temperature of approximately 18.3 °C and a mean annual precipitation of about 556 mm, accompanied by pronounced seasonal variability. Relative humidity averages around 70%, while annual potential evaporation exceeds 1400 mm. Prior to establishing the experiment, soil samples were collected to characterize the physical and chemical properties of the site. Particle-size distribution was determined using the pipette method in compliance with ISO 11277:2009, revealing a heavy textured profile dominated by clay and silty clay fractions. Soil water retention characteristics were quantified using a pressure plate extractor at -33 kPa and -1500 kPa matric potentials, yielding field capacity and permanent wilting point values of 384 mm and 273 mm, respectively, within the 0–60 cm soil layer (ISO, 2009; Dane and Topp, 2002). Bulk density was measured on undisturbed cores following USDA-NRCS soil quality guidelines, and values ranged between 1.34 and 1.43 g cm^-3^ (USDA-NRCS, 2004). Soil pH and electrical conductivity (EC_1:1_) were determined in a 1:1 soil to water suspension using a digital multiparameter benchtop meter, based on standardized laboratory procedures, resulting in pH values of 7.8-8.1 and EC values ranging from 0.40 to 0.50 dS m^-1^ (USDA-NRCS, 2014).

### 2.2 Experimental design and irrigation management

The calibration and validation procedures were carried out following the standard protocol recommended for process-based crop simulation models. Model calibration was performed using measurements from the 2024 growing season, while validation was conducted using data from the 2025 season under the same experimental conditions and irrigation treatments. The field experiment was established using a randomized split-plot design with four replications, incorporating three irrigation regimes: I_100_, representing full irrigation in which the soil water deficit within the 0–60 cm profile was replenished to field capacity at a 7-day interval; I_70_, where 70% of the I_100_ irrigation amount was applied; and I_50_, where irrigation was limited to 50% of the full irrigation requirement. Each subplot measured 10 m in length and 4.2 m in width (42 m²).

A surface drip irrigation system was installed in all plots, with laterals installed along each crop row. The emitters had a flow rate of 1.8 L h^-1^ at the operating pressure and were spaced at 40 cm intervals on drip lines with a wall thickness of 1 mm. Irrigation water was supplied from a deep well located at the experimental site. Filtration was provided by a combined hydrocyclone and disk filter unit with inlet and outlet diameters of 90 mm, ensuring adequate removal of suspended particles. The system operated at a constant pressure of 150 kPa throughout the irrigation period to maintain uniform discharge along the laterals.

### 2.3 Crop establishment and agronomic management

In both growing seasons, the soybean cultivar Turbo (Glycine max (L.) Merr.) was grown under field conditions. Planting was carried out on 22 June in both 2024 and 2025, with seeds sown at a depth of 5 cm and a target plant density of 200 plants m^-^². Manual harvesting was performed on 10 October 2024 and 23 October 2025. Fertilization practices were standardized across treatments, and nutrient requirements were determined based on preplant soil analyses. At sowing, all plots received 180 kg ha^-^¹ of diammonium phosphate (DAP, 18-46-0) as the initial fertilizer input.

### 2.4 Crop and climate modeling framework

Crop simulation models are widely used to analyze how climate variability and other environmental factors affect crop growth and yield, providing an important framework for assessing future agricultural performance. By representing the physiological processes that regulate plant development, these models can realistically capture temporal growth dynamics and provide a valuable basis for projecting yield responses under future conditions (White et al., 2011; Huang et al., 2018). Among these tools, the Cropping System Model within the DSSAT framework can represent crop development, biomass accumulation, and yield responses under changing weather, soil properties, and management practices (Jones et al., 2003). The DSSAT-CSM operates at the point scale, integrating cultivar genetics, water and nutrient dynamics, and seasonal management to simulate daily plant processes across single or multiple cropping seasons. It has been applied across diverse production systems, where researchers have used the model to examine climate change impacts at varying spatial scales and to assess the performance of cropping systems under contrasting conditions and management strategies (Carbone et al., 2003; Sarkar and Kar, 2006; Yakoub et al., 2017).

Projections generated by global and regional climate models inherently involve considerable uncertainty, largely due to limitations in spatial resolution and resulting inconsistencies among model outputs. Consequently, many studies rely on ensembles derived from several climate models to improve the robustness and reliability of future climate assessments (Fischer et al., 2005; Ranjitkar et al., 2016). Future climate conditions are commonly estimated using Global Climate Models (GCMs) which numerically represent large-scale atmospheric and oceanic processes as well as interactions within the Earth system (IPCC, 2013). These models represent the most sophisticated tools available for assessing how the global climate responds to changes in greenhouse gas concentrations and are therefore fundamental to contemporary climate change research (Melo et al., 2015).

Although the methodological framework adopted in this study relies on well-established modeling tools, the DSSAT-CROPGRO-Soybean model was selected because it enables the integrated simulation of crop phenology, canopy development, biomass accumulation, and grain yield under varying climatic and water availability conditions. In addition, the DSSAT modeling framework allows the incorporation of different atmospheric CO_2_ concentrations into the simulation environment, enabling the assessment of crop responses under future climate scenarios characterized by elevated CO_2_ levels. Furthermore, the use of multiple GCM projections rather than a single climate model was preferred in order to better represent the uncertainty associated with future climate conditions. Compared with studies relying on a single climate projection, this multi-GCM approach provides a broader and more robust basis for evaluating potential soybean yield responses under different irrigation regimes and climate scenarios.

In this study, future climate projections were derived from three global climate models (HadGEM2-ES, MPI-ESM-MR, and GFDL-ESM2M) selected from the CMIP5 model ensemble (Taylor et al., 2012). The corresponding datasets, produced for both emission pathways, were obtained from the Turkish State Meteorological Service (TSMS) which generated high-resolution outputs by dynamically downscaling each model using the RegCM4.3.4 regional climate framework at a spatial resolution of 20 km.

A bias correction procedure was implemented to minimize systematic differences between simulated and observed climate conditions for the historical baseline (reference) period (1970-2000). Daily outputs from each climate model were adjusted independently for all variables using station observations from the study area, thereby correcting mean biases and improving the consistency of temperature and precipitation fields. These adjustments improved the suitability of the climate inputs for DSSAT simulations. After bias correction, projected climatic conditions were evaluated across three future assessment periods 2016-2040 (near future), 2041-2070 (mid-century) and 2071-2098 (late-century) each compared with the reference climatology of 1970–2000 to quantify the magnitude and direction of climate-driven changes.

Climate research commonly relies on standardized greenhouse gas concentration pathways, known as Representative Concentration Pathways (RCPs), which were formulated to ensure consistency in scenario development for global and regional climate modeling (IPCC, 2014). These pathways, RCP 2.6, RCP 4.5, RCP 6.0 and RCP 8.5 are distinguished, by the magnitude of radiative forcing they are projected to reach by 2100, reflecting the progression of greenhouse gas concentrations from the present day toward the end of the century (Meinshausen et al., 2011). In its Fifth Assessment Report (AR5), the IPCC also underscored the need to use downscaled outputs from global climate models to improve the regional relevance of climate impact assessments.

Representative atmospheric CO_2_ concentrations were assigned to each future period according to the corresponding RCP pathway in the study. For RCP 4.5, the mean concentrations applied in the DSSAT simulations were approximately 436 ppm for the near-future period (2016-2040), 509 ppm for the mid-century period (2041-2070), and 534 ppm for the late-century period (2071-2098). Under RCP 8.5, the respective values were higher, with average concentrations of about 450 ppm, 593 ppm and 846 ppm. The historical baseline simulations were based on a representative CO_2_ level of approximately 347 ppm, consistent with measurements reported for the 1970–2000 period (NOAA Global Monitoring Laboratory, 2023). These concentrations were incorporated directly into the DSSAT model by specifying the appropriate CO_2_ value for each scenario and time period prior to running the simulations.

### 2.5 Model calibration and validation

A field experiment conducted during the 2024–2025 growing seasons provided the data for calibrating and validating the DSSAT-CROPGRO-Soybean model. Calibration was performed using field-observed phenological stages and growth parameters, which were used to derive the genetic coefficients required to accurately represent the cultivar’s growth and development in the study region. Key phenological indicators, including emergence (EDAT), first flowering (anthesis) (ADAT), first pod stage (PD1T), physiological maturity (MDAT), canopy height (CHTA) and maximum LAI (LAIX) were monitored under three irrigation regimes (I_100_, I_70_ and I_50_). The observed phenological dates were then entered into the DSSAT experimental files (FileA and FileT), allowing iterative adjustment of the genetic coefficients until satisfactory agreement was achieved between simulated and observed values. However, the DSSAT guidelines recommend at least 6000 runs for GLUE (Jones et al., 2011), in this study 20.000 runs were generated using the phenology only option on a high-performance computer. In order to obtain more accurate coefficient values, the results were manually checked and adjusted, where necessary, following DSSAT guidelines.

Model validation was conducted using an independent data set, where simulated values were compared against measured leaf area index (LAI), aboveground biomass, and yield. LAI measurements were taken biweekly using an optical canopy analyzer (LAI-2000), whereas biomass was quantified through destructive sampling by collecting plants from a defined 1 m² area in each plot. Samples were oven dried at 70 °C to constant weight to determine dry biomass.

### 2.6 Statistical evaluation of model performance

To evaluate the performance of the CROPGRO-Soybean simulations in both the calibration and validation phases, two complementary diagnostic approaches were applied to different groups of variables. Taylor diagrams were used to assess the agreement between observed and simulated values of grain yield, aboveground biomass and leaf area index (LAI), by jointly summarizing correlation, relative variance and centered root mean square deviation (RMSD) for these key growth and productivity indicators. In the Taylor diagram analysis, the centered RMSD was calculated as.


$$RMSD=\sqrt{\frac{1}{n}\sum_{i=1}^{n}[{\left((S_{i}-\bar{S})-(O_{i}-\bar{O})\right)}^{2}]}$$

where $S_{i}$ and $O_{i}$ denote simulated and observed values, respectively, $\bar{S}\;$ and $\bar{O}\;$ are their means, and n  is the number of paired observations. In parallel, a heatmap-based error analysis was conducted for the phenological and canopy-related variables, including emergence date (EDAT), anthesis date (ADAT), pod development timing (PD1T), physiological maturity date (MDAT), canopy height (CHTA) and maximum leaf area index (LAIX). This approach allowed the magnitude and pattern of deviations to be visualized across irrigation treatments and measurement dates. The combined use of Taylor diagrams for yield and growth-related variables and heatmaps for phenological and structural attributes provided a comprehensive evaluation framework that links field observations to model behavior and supports the reliability of the calibrated model for subsequent climate scenario simulations.

## 3 Results

### 3.1 Evaluation of model performance during calibration and validation stages

**Table 1** summarizes the default genetic coefficients included in DSSAT-CROPGRO-Soybean and the calibrated values derived using the Generalized Likelihood Uncertainty Estimation (GLUE) software. The GLUE-calibrated parameter set provided the best fit to the observed phenological and growth measurements under the environmental conditions of the study area. Although the GLUE was used during the calibration stage, manual adjustments were applied when the optimization process did not achieve an adequate fit to the observed data.

**Table 1:** Comparison of default and GLUE-calibrated genetic coefficients for soybean.

Soybean genetic coefficient	Description of the coefficient	Default coefficient values	Calibrated coefficient values
LFMAX	Maximum leaf photosynthesis rate at 30 °C, 350 ppm CO_2_, and high light (mg CO_2_/m^-2^s^-1^)	1.030	2.675
SLAVR	Specific leaf area of cultivar under standard growth conditions (cm^2^ g^-1^)	385	690
SIZLF	Maximum size of full leaf (three leaflets) (cm^2^)	137.0	160.0
XFRT	Maximum fraction of daily growth that is partitioned to seed + shell	1.00	1.00
WTPSD	Maximum weight per seed (g)	0.155	0.100
SFDUR	Seed filling duration for pod cohort at standard growth conditions (photothermal days)	22.0	14.0
SDPDV	Average seed per pod under standard growing conditions (number per pod)	2.20	2.20
PODUR	Time required for cultivar to reach final pod load under optimal conditions (photothermal days)	7.5	1.5
THRSH	Threshing percentage. The maximum ratio of (seed/(seed+shell)) at maturity. Causes seeds to stop growing as their dry weight increases until shells are filled in a cohort.	78.0	95.5
SDPRO	Fraction protein in seeds (g(protein)/g(seed))	0.400	0.200
SDLIP	Fraction oil in seeds (g(oil) g(seed)^-1^)	0.400	0.200

**Figure 1:**
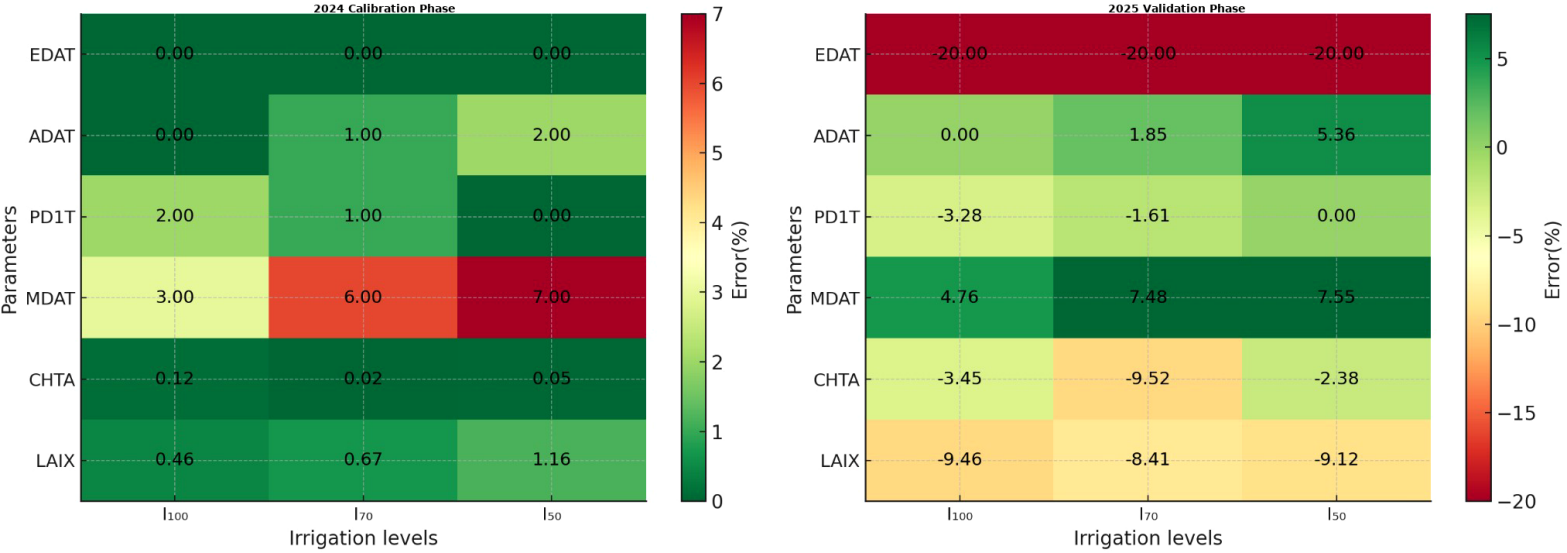
Absolute error heatmap of phenological-stage calibration (2024) and validation (2025) under varying irrigation conditions.

**Figure 1** illustrates the 2024 calibration results for phenological and morphological parameters across irrigation treatments. The analysis of phenological and morphological errors showed that the calibrated DSSAT model reproduced field observations with only limited deviations. According to the heatmap, the smallest deviations were consistently observed in the early phenological stages across all irrigation levels, particularly for EDAT (emergence date) and ADAT (anthesis date) under full irrigation (I_100_), where the simulations closely matched the measured values. PD1T also showed limited deviation under I_100_ and I_70_, with only slight increases in error under the lowest irrigation level (I_50_). The largest deviations were observed with the MDAT (maturity date) stage; however, these values remained within acceptable ranges. Morphological indicators exhibited a similar trend: canopy height (CHTA) and maximum leaf area index (LAIX) were reproduced with high precision under all irrigation regimes, and although minor increases in error were observed under deficit irrigation, the overall deviations remained limited.

**Figure 1** also presents the model validation results using the 2025 field dataset. The validated model reproduced field observations with limited deviations across irrigation treatments. According to the heatmap, the smallest deviations were consistently observed in the early phenological stages across all irrigation levels, particularly for EDAT and ADAT under full irrigation (I_100_), where the simulations closely matched the measured values. PD1T also showed limited deviation under I_100_ and I_70_, with slightly increased errors under the I_50_ treatment. The largest deviations were observed at the MDAT stage; however, these values remained within acceptable limits. Morphological variables, including CHTA and LAIX, also showed high agreement between simulated and observed values under all irrigation regimes. Although modest deviations were evident under deficit irrigation conditions, the overall error remained limited.

**Figure 2:**
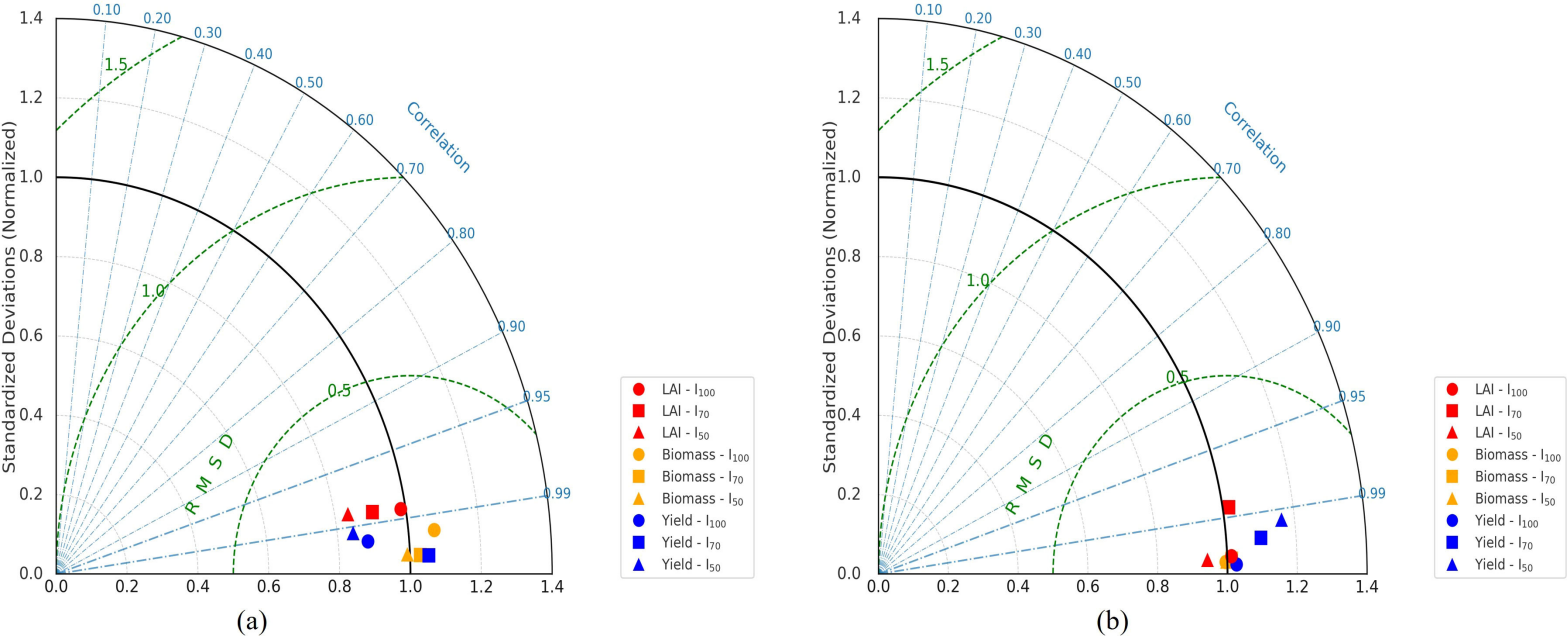
Taylor diagram assessing DSSAT-CROPGRO-Soybean performance for the 2024 calibration **(a)** and 2025 validation **(b)**.

The results of the 2024 model calibration (a) and the 2025 validation (b) are presented in **Figure 2** using a Taylor diagram. The diagram illustrates the statistical agreement between observed and simulated leaf area index (LAI), biomass, and grain yield under full (I_100_), moderate (I_70_), and deficit (I_50_) irrigation conditions. During the 2024 calibration period, correlation coefficients showed strong agreement agreement across all three variables and irrigation treatments, while the normalized standard deviations closely matched the reference values. Most RMSD contours were below 1.0, indicating close agreement between observed and simulated values. **Table 2** shows that the corresponding R² values were also high during calibration, particularly under I_100_ and I_70_ treatments.

**Table 2:** Statistical performance indicators of the DSSAT-Cropgro model during calibration (2024) and validation (2025) stages.

Stages	Variables	Correlation			R^2^			RMSD		
		I_100_	I_70_	I_50_	I_100_	I_70_	I_50_	I_100_	I_70_	I_50_
Calibration	LAI	0.92	0.91	0.89	0.85	0.83	0.79	0.41	0.44	0.49
	Biomass	0.94	0.92	0.91	0.88	0.85	0.83	0.35	0.41	0.44
	Yield	0.93	0.90	0.86	0.87	0.81	0.75	0.38	0.47	0.56
Validation	LAI	0.99	0.99	0.99	0.99	0.97	0.98	0.04	0.17	0.07
	Biomass	1.00	0.99	0.98	0.99	0.99	0.97	0.03	0.10	0.22
	Yield	1.00	0.99	1.00	0.99	0.98	0.98	0.02	0.67	0.07

The simulated LAI closely matched with observed values, particularly under full and moderate irrigation conditions. Under I_100_ and I_70_ treatments, the LAI points clustered in regions characterized by high correlation and low RMSD. Under the deficit irrigation treatment (I_50_), a slight increase in RMSD was observed; however, the correlation coefficient remained high. Biomass simulations were in strong agreement agreement with measured values. Under I_100_ and I_70_ irrigation teratments, biomass points were positioned close to the reference standard deviation arc. Under I_50_ conditions, RMSD values increased slightly but remained within acceptable limits. Grain yield simulations during the calibration phase also closely reproduced the observed values, especially under I_100_ and I_70_ irrigation treatments. Yield points clustered near the reference point with RMSD values mostly below 0.5. Under the I_50_ treatment, small increases in RMSD were observed; nevertheless, the correlation coefficients remained high.

The 2025 validation results also indicated high agreement between observed and simulated datasets. For all validation datasets, correlation coefficients for LAI, biomass, and grain yield remained high, while RMSD values generally stayed below 1.0. In addition, the R² values presented in **Table 2** further confirmed the strong consistency between observed and simulated values during the validation stage, with the highest agreement generally obtained under I_100_ conditions.

### 3.2 Bias-corrected outputs of three GCMs under RCP 4.5 and RCP 8.5 scenarios

**Table 3** summarizes the results of the bias correction applied to three global climate models (GFDL-ESM2M, HadGEM2-ES, MPI-ESM-MR) under the RCP 4.5 and RCP 8.5 pathways, based on comparisons with the 1970–2000 baseline (reference) period. The bias-corrected climate projections reveal a consistent warming pattern across all three global climate models and both emission pathways. For maximum temperatures, GFDL-ESM2M exhibits a progressive increase from approximately 6.9–7.1 °C in 2016–2040 to nearly 7.5–9.7 °C by the 2071–2098 period. HadGEM2-ES presents a similar upward response, rising from approximately 5.5–5.8 °C in the early decades to 6.8–8.7 °C toward the end of the century. MPI-ESM-MR shows more moderate increase, with Tmax increasing from about 4.4 °C to 5.2–7.7 °C over the same periods.

**Table 3:** Bias corrected changes in Tmax, Tmin, and precipitation for three GCMs under RCP 4.5 and RCP 8.5 across future periods.

GCMs	Periods	ΔTmax (°C)		ΔTmin (°C)		Precipitation change (mm)	
		RCP 4.5	RCP 8.5	RCP 4.5	RCP 8.5	RCP 4.5	RCP 8.5
GFDL-ESM2M	2016-2040	6.92	7.09	4.51	4.75	1.62	0.68
	2041-2070	7.28	8.21	4.92	5.68	1.00	0.56
	2071-2098	7.47	9.68	5.07	6.95	0.62	0.08
HadGEM2-ES	2016-2040	5.51	5.81	3.94	4.31	-0.25	-0.22
	2041-2070	6.24	6.99	4.54	5.33	-0.56	-0.50
	2071-2098	6.83	8.73	5.16	7.08	-0.29	-0.24
MPI-ESM-MR	2016-2040	4.38	4.35	2.93	2.95	0.34	0.29
	2041-2070	4.66	5.57	3.25	3.91	0.38	0.32
	2071-2098	5.16	7.65	3.61	5.64	0.34	0.03

Tmin and Tmax denote the period means of daily minimum and daily maximum air temperature (°C), respectively, expressed as changes relative to the baseline period, while precipitation changes are reported as daily averages (mm), i.e., mm per day, relative to the baseline.

Minimum temperatures follow a similar trend: GFDL-ESM2M indicates an increase from around 4.5–4.8 °C to 5.1–7.0 °C, HadGEM2-ES from 3.9–4.3 °C to 5.2–7.1 °C, and MPI-ESM-MR from 2.9–3.0 °C to 3.6–5.6 °C between the earliest and latest projection periods. Precipitation anomalies vary by model relative to the baseline period. For GFDL-ESM2M, the projected precipitation change remains positive but weakens over time, decreasing from 1.62–0.68 mm in 2016–2040 to 0.62–0.08 mm in 2071–2098. HadGEM2-ES remains consistently negative, ranging from −0.25 to −0.56 mm, while MPI-ESM-MR indicates minor reductions, declining from approximately 0.29–0.38 mm to 0.34–0.03 mm through the projection horizon.

Overall, warming was more pronounced under RCP 8.5 under RCP 8.5, with GFDL-ESM2M showing the highest temperature anomalies and MPI-ESM-MR the lowest. The drying trend observed in the precipitation projections was also more pronounced under RCP 8.5.

### 3.3 Temperature and precipitation trends across three projection periods under RCP 4.5 and RCP 8.5 scenarios

**Figure 3:**
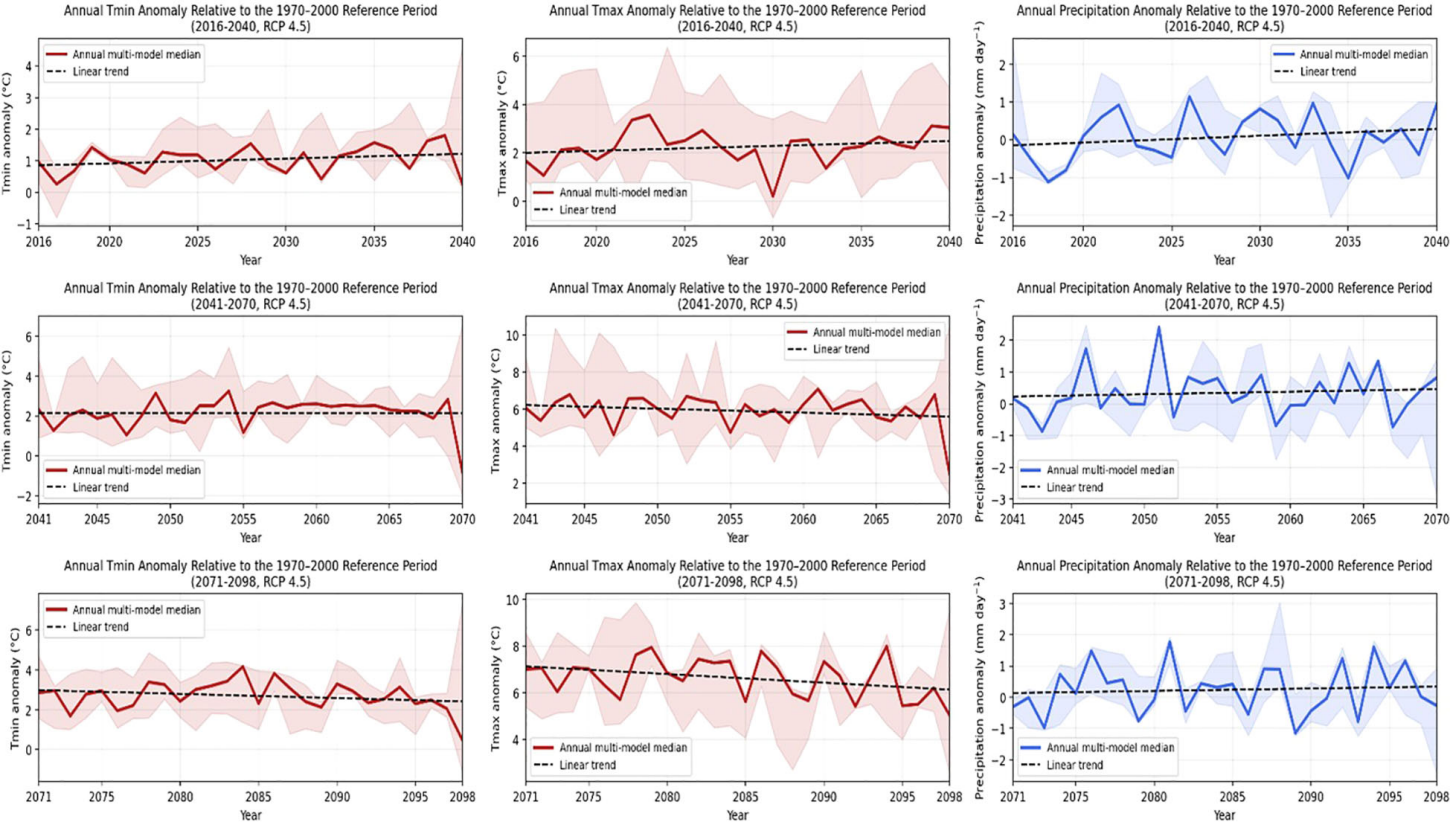
Annual projections of temperature (Tmin and Tmax) and precipitation anomalies under the RCP 4.5 scenario for the periods 2016–2040, 2041–2070, and 2071–2098 relative to the 1970–2000 baseline. The shaded area represents the inter-model range (minimum–maximum) across the three GCMs (GFDL-ESM2M, HadGEM2-ES, and MPI-ESM-MR), while the solid line indicates the annual multi-model median. The dashed line shows the linear trend of the multi-model median.

**Figure 4:**
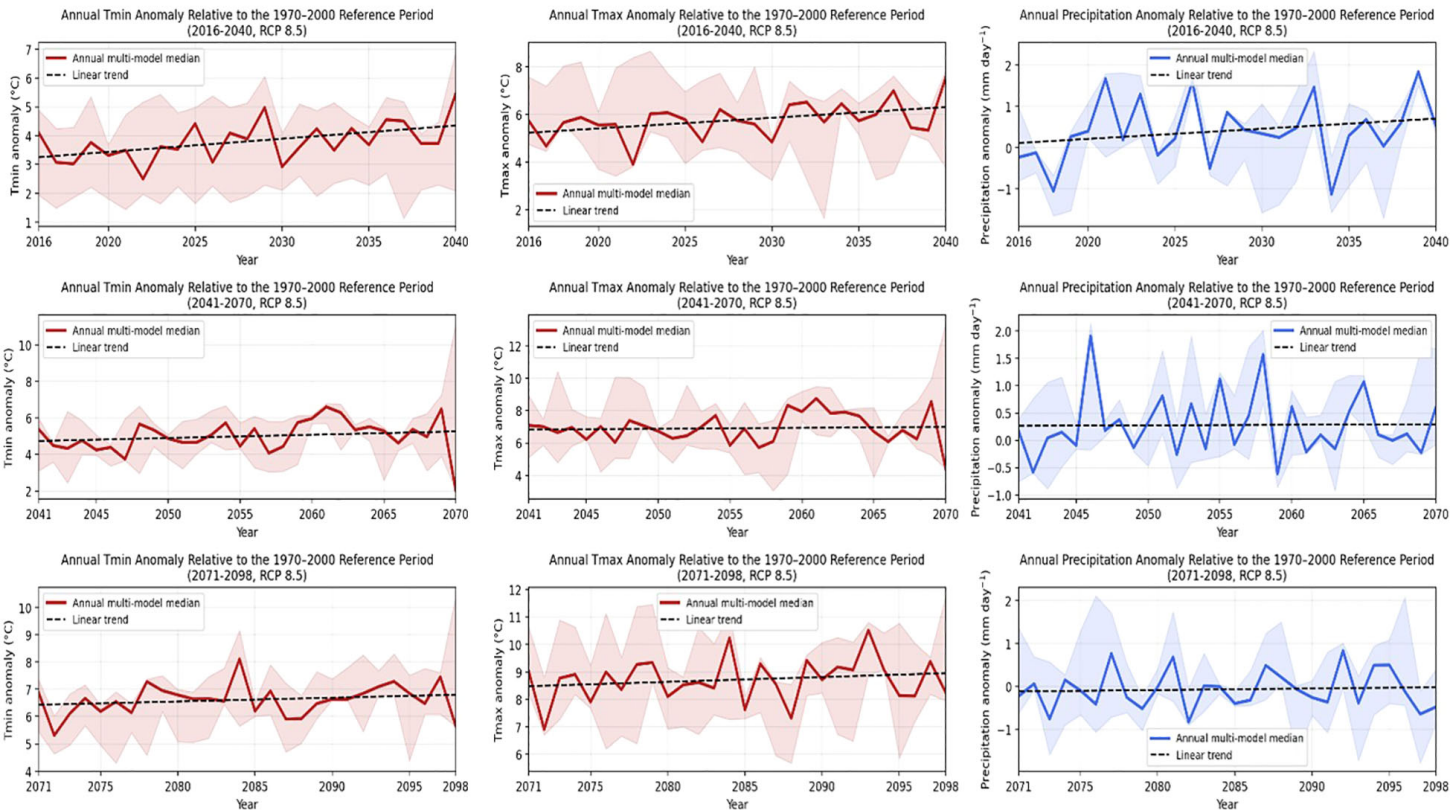
Annual projections of temperature (Tmin and Tmax) and precipitation anomalies under the RCP 8.5 scenario for the periods 2016–2040, 2041–2070, and 2071–2098 relative to the 1970–2000 baseline. The shaded area represents the inter-model range (minimum–maximum) across the three GCMs (GFDL-ESM2M, HadGEM2-ES, and MPI-ESM-MR), while the solid line indicates the annual multi-model median. The dashed line shows the linear trend of the multi-model median.

Annual temperature and precipitation anomalies under the RCP 4.5 and RCP 8.5 emission pathways were derived from three global climate models (GFDL-ESM2M, HadGEM2-ES, and MPI-ESM-MR) and visualized using shaded model envelopes and multi-GCM median lines. In **Figures 3**, **4**, the shaded area represents the inter-model range (minimum–maximum) of the annual projections, whereas the solid line denotes the annual multi-GCM median. The results for the RCP 4.5 scenario are presented in **Figure 3**, indicating a consistent and persistent warming signal across all projection periods. Both Tmin and Tmax display steadily increasing anomalies with relatively narrow envelopes during 2016–2040, indicating strong agreement among models. In contrast, precipitation anomalies fluctuate around near-neutral values and exhibit substantially higher interannual variability. During the mid-century period (2041–2070), the shaded envelopes widen, particularly for Tmax, as inter-model divergence increases in parallel with a strengthening climate signal. Temperature anomalies continue to increase, whereas precipitation responses show modest increases but remain heterogeneous. By the late-century period (2071–2098), Tmin and Tmax anomalies reach their highest levels, while precipitation projections continue to show notable spread.

**Figure 4** presents the annual temperature and precipitation anomalies under the RCP 8.5 pathway. Compared with RCP 4.5, the RCP 8.5 projections display a more pronounced warming signal beginning in the near-future period (2016–2040). Both Tmin and Tmax anomalies exhibit a steeper positive trajectory, with wider envelopes indicating increased divergence among models as radiative forcing intensifies. Precipitation anomalies fluctuate around near-neutral values but with substantially higher interannual variability. In the mid-century (2041–2070) and late-century (2071–2098) periods, the warming trend becomes significantly stronger, and the shaded envelopes expand further, particularly for maximum temperatures.

The annual multi-GCM median of Tmin and Tmax rises to elevated ranges, while precipitation responses remain highly variable and do not converge toward a common signal. Overall, the RCP 8.5 results indicate stronger thermal stress and greater climatic variability relative to RCP 4.5.

### 3.4 Climate scenario impacts on future soybean yields

Soybean yield projections varied considerably across emission pathways, projection periods, irrigation regimes, and GCMs. Across the evaluated simulations, the contrast between RCP 4.5 and RCP 8.5 became more evident in the late-century period, and this difference was consistently stronger under irrigated conditions than under rainfed conditions. In general, the higher atmospheric CO_2_ concentrations associated with RCP 8.5 contributed to higher simulated yields than those under RCP 4.5, although the magnitude of this response depended strongly on water availability and model-specific climate projections.

**Figure 5:**
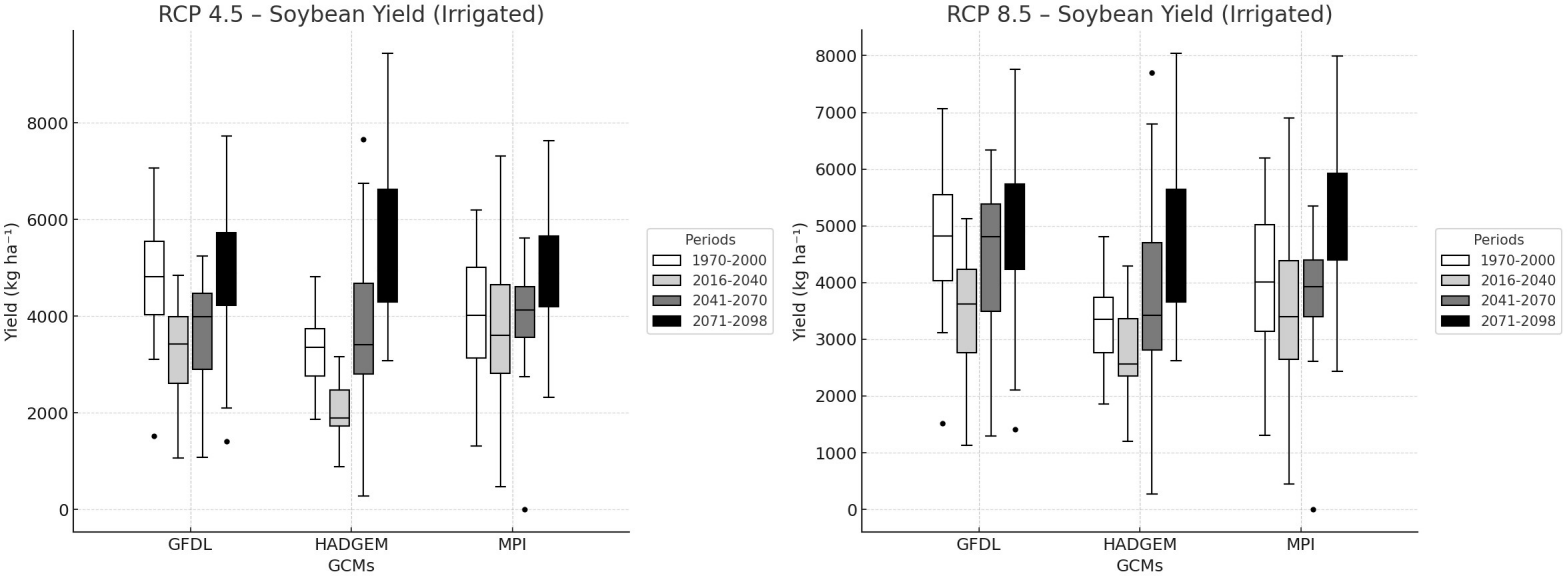
Irrigated soybean yield projections under RCP 4.5 and RCP 8.5 across future periods.

Under irrigated conditions (**Figure 5**), soybean yields were consistently higher under RCP 8.5 than under RCP 4.5 across all future periods and GCMs, with the clearest separation observed in the late-century period (2071–2098). In GFDL-ESM2M, late-century irrigated yield increased from approximately 5200 kg ha^-^¹ under RCP 4.5 to about 5600 kg ha^-^¹ under RCP 8.5, corresponding to an increase of approximately 8–10%. In HadGEM2-ES, the increase was smaller, from around 4800 to 5000 kg ha^-^¹, representing about 4–6%. The largest difference between scenarios was observed in MPI-ESM-MR, where yield increased from approximately 4500 kg ha^-^¹ under RCP 4.5 to nearly 5200 kg ha^-^¹ under RCP 8.5, equivalent to about 15–17%. These results indicate that the positive effect of elevated CO_2_ was translated more effectively into yield gains when irrigation prevented severe water limitation.

Irrigated yield patterns over time also differed between scenarios. Under RCP 4.5, yields increased gradually from the near future to the end of the century. Under RCP 8.5, however, the increase became more pronounced during the mid-century and late-century periods, leading to a stronger upward shift in median yield trajectories. The widening gap between the lower and upper bounds of the model envelope, especially under HadGEM2-ES and GFDL-ESM2M, further indicates that inter-model uncertainty increased over time, particularly under the higher-emission scenario.

**Figure 6:**
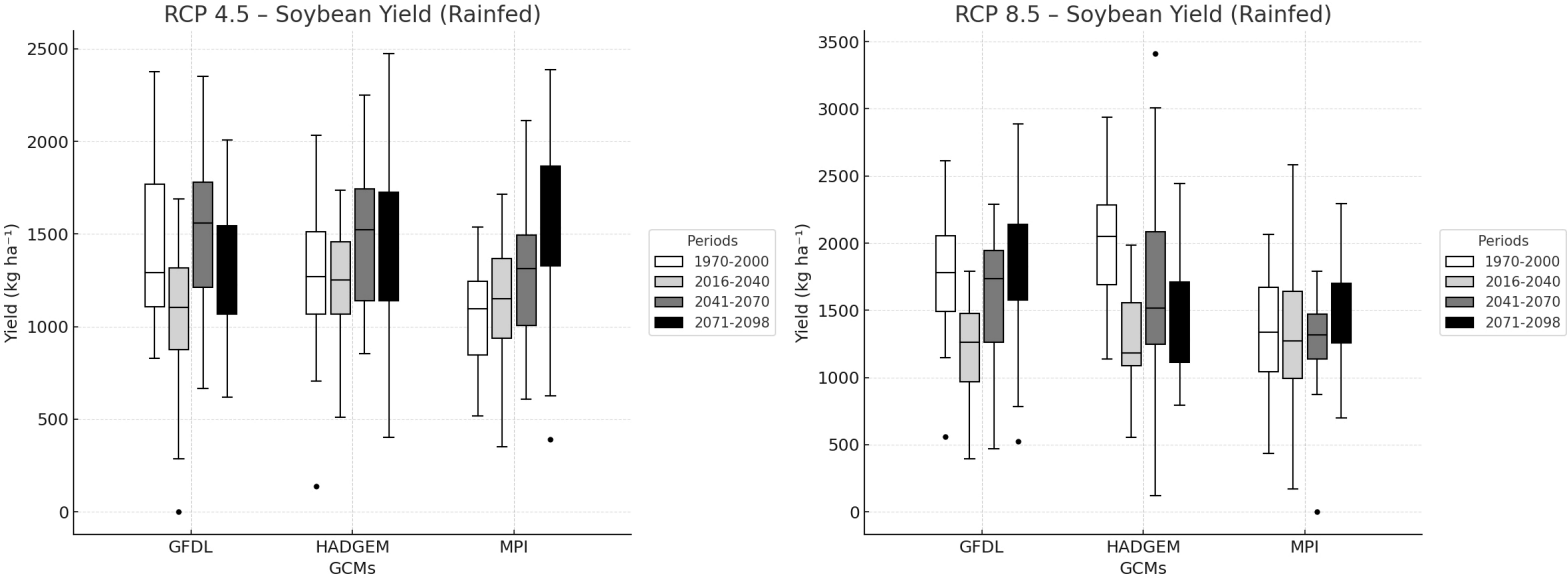
Rainfed soybean yield projections under RCP 4.5 and RCP 8.5 across future periods.

Under rainfed conditions (**Figure 6**), the difference between RCP 4.5 and RCP 8.5 remained smaller and more variable than those under irrigation, despite the generally higher CO_2_ concentrations under RCP 8.5. In GFDL-ESM2M, yield increased from approximately 1500 kg ha^-^¹ under RCP 4.5 to about 1800 kg ha^-^¹ under RCP 8.5, corresponding to an increase of nearly 20%. In HadGEM2-ES, the difference was more limited, with yield increasing from about 1600 to 1700 kg ha^-^¹ (approximately 6–8%). In MPI-ESM-MR, yields increased from approximately 1400 to 1600 kg ha^-^¹, corresponding to about 14–16%. Although RCP 8.5 still produced higher yields than RCP 4.5 in most cases, the absolute yield levels remained substantially below those of irrigated systems throughout all projection periods.

Among the three GCMs, GFDL-ESM2M showed the highest increase in temperature across the projection periods, indicating the most extreme warming conditions for the study region. In contrast, the greatest difference in irrigated soybean yield projections between RCP 4.5 and RCP 8.5 was found in MPI-ESM-MR, whereas HadGEM2-ES showed the smallest difference.

A key finding of the rainfed analysis is that the beneficial effect of elevated CO_2_ did not overcome the dominant influence of seasonal water limitation. Relative to irrigated conditions, rainfed yields remained lower, more variable, more strongly dependent on model differences, indicating that soil moisture availability remained the main constraint on crop productivity. Overall, **Figures 5**, **6** indicate that future yield responses were shaped by the interaction of atmospheric CO_2_ enrichment, warming intensity, and water supply, with irrigation determining whether the positive physiological response to elevated CO_2_ could be expressed as a yield advantage.

## 4 Discussion

The calibrated DSSAT-CROPGRO-Soybean model reproduced soybean phenology, canopy development, biomass accumulation, and grain yield with strong agreement across irrigation treatments. The low deviations observed for EDAT, ADAT, CHTA, LAIX, LAI, biomass, and yield indicate that the calibration procedure successfully captured the local crop response. Similar levels of agreement have been reported in previous DSSAT-based studies for soybean and other crops under contrasting irrigation regimes and environmental conditions, confirming that the model framework can capture growth dynamics with satisfactory accuracy when cultivar coefficients are properly calibrated (Akumaga et al., 2023; Bebeley et al., 2022; Zhang et al., 2024; Tooley et al., 2021). The larger deviations observed at MDAT in both calibration and validation likely reflect the greater sensitivity of maturity timing to accumulated thermal stress and late-season water limitation, which is also consistent with previous crop modeling studies (Salmeron and Purcell, 2016; Nargund et al., 2024).

The Taylor diagram analysis provided further support for the model’s reliability. High correlation coefficients, low RMSD values, and close agreement between simulated and observed standard deviations indicated that the model reproduced the temporal patterns of LAI, biomass, and grain yield across irrigation levels. The slight increase in RMSD under deficit irrigation suggests that water stress introduced greater variability, but the model still maintained reliable predictive performance. Comparable findings have been reported in other DSSAT-based studies where stress conditions increased deviations without substantially reducing the overall agreement between measured and simulated seasonal patterns (Kassie et al., 2016; Mohammed and Feleke, 2022). These results support the use of the calibrated model for evaluating soybean responses under future climate and irrigation scenarios.

The projected climate projections indicated progressive warming under both emission pathways, with stronger temperature increases and a more pronounced drying trend under RCP 8.5. The higher anomalies simulated from GFDL-ESM2M and the more moderate anomalies from MPI-ESM-MR are in line with previous regional studies showing that projections for the Mediterranean region and Türkiye differ substantially across climate models, particularly for precipitation (Demircan et al., 2017; Öztürk et al., 2015; Giorgi and Lionello, 2008). The wide spread among precipitation projections projections also reflects the well-known uncertainty of hydrological outputs in this region. Therefore, the use of multiple GCMs was important for representing the range of possible future climatic conditions rather than relying on a single climate model outcome.

Under irrigated conditions, soybean yields generally increased in future periods, especially under RCP 8.5 and in the late-century interval. This pattern suggests that elevated atmospheric CO_2_ partly offset the adverse effects of warming when water supply was maintained at adequate levels. Similar responses have been reported in previous soybean studies, in which irrigation and CO_2_ enrichment together enhanced biomass production and seed yield under future climate scenarios (Fernandes et al., 2022; Bigolin and Talamini, 2025; Li et al., 2024). However, the magnitude of response differed among GCMs. MPI-ESM-MR showed the largest difference between RCP 4.5 and RCP 8.5 despite not generating the strongest warming signal, indicating that yield outcomes were shaped not only by temperature but also by the interaction between CO_2_ concentration, seasonal precipitation patterns, and soil water balance.

In contrast, rainfed soybean remained substantially more vulnerable to future climatic stress. Although RCP 8.5 sometimes produced higher yields than RCP 4.5, the absolute yield under rainfed conditions remained much lower than under irrigation. This indicates that the fertilization effect of elevated CO_2_ was not sufficient to overcome moisture limitations. Similar constraints have been reported in soybean and maize studies under water-limited conditions, where drought stress, increased evaporative demand, and shortened reproductive phases restricted the potential benefits of elevated CO_2_ (MacCarthy et al., 2022; Sen et al., 2023, 2024). The persistence of a wide irrigated–rainfed yield gap across all periods in the present study confirms that seasonal soil water availability remains the dominant limiting factor in soybean production under Mediterranean conditions.

Differences among GCMs introduced additional uncertainty into future soybean projections. GFDL-ESM2M generally generated stronger warming and lower moisture availability, resulting in greater stress on rainfed production, whereas MPI-ESM-MR produced more moderate climatic changes and relatively stable yield responses during some projection periods. This inter-model variability supports the need for ensemble-based climate impact assessments, particularly in semi-arid Mediterranean regions where precipitation changes remain highly uncertain.

Although the model performance was strong, several uncertainties remain. The simulations were based on projected temperature, precipitation, and atmospheric CO_2_ concentrations, while other possible stressors such as heatwave frequency, pest pressure, and nutrient constraints were not explicitly represented. In addition, local management adaptations other than irrigation were not considered in the scenario analysis. Even so, the combined calibration–validation framework together with the multi-scenario assessment provide a reliable basis for evaluating climate-related risks and supporting adaptation-focused irrigation planning for soybean production in Mediterranean environments.

## 5 Conclusion

These findings underscore the critical role of irrigation in stabilizing soybean production under changing climatic conditions. The model accurately represented phenology, growth dynamics, and yield across multiple irrigation regimes, supporting its suitability for evaluating climate-driven risks. Scenario simulations indicated that irrigated soybean production is likely to maintain, and in some scenarios improve, yield potential under elevated CO_2_ levels, whereas rainfed conditions are expected to remain more vulnerable, with greater yield variability due to stronger temperature stress and increased evaporative demand.

These findings underscore the critical role of irrigation in stabilizing soybean production under climate change. They also demonstrate the value of multi-GCM climate assessments for quantifying uncertainty and guiding regional water management strategies. Effective adaptation will require the optimization of irrigation scheduling, improvement of water use efficiency, and careful monitoring of climatic thresholds affecting reproductive development.

Despite the satisfactory performance of the model, several limitations of the present study should be acknowledged. (i) The determination of crop genetic coefficients remains an important limitation. Although the GLUE-based procedure within the model estimates genetic coefficients by comparing simulated outputs with field observations, some limitations still remain. In this context, during the calibration process, determining coefficients based on plant growth parameters and phenological stages may lead to the estimation of different genetic coefficients. (ii) When stress factors such as water deficit are considered during the model calibration and validation stages, large differences may occur between model outputs and field observations. Therefore, during calibration and validation, greater emphasis should be placed not only on stress factors but also on management-related variables such as varying sowing dates and planting densities. (iii) The DSSAT model requires daily climatic inputs. Low-quality meteorological data obtained from weather stations, or the presence of missing observations, may limit the predictive performance of the model. (iv) The integration of artificial intelligence into crop simulation models could offer important benefits. In climate change studies, artificial intelligence could be used to retrieve data such as CO_2_ concentrations from official web-based sources and integrate them directly to users within the modeling framework. In particular, the presence of an automatic weather station in the study region and the ability to automatically integrate these data into the model could provide a significant advantage. In addition, with such artificial intelligence support, errors that occur during model operation could be identified and resolved more efectively. Furthermore, the model could also be enhanced through AI-supported automation. Machine learning algorithms, real-time sensor networks, and autonomous decision-support tools can enhance model calibration, improve forecasting accuracy, and enable dynamic irrigation management that responds to real-time plant-soil-atmosphere feedback. (v) In this study, annual temperature and precipitation anomalies under the RCP 4.5 and RCP 8.5 emission pathways were derived from three global climate models (GFDL-ESM2M, HadGEM2-ES, and MPI-ESM-MR) and visualized using shaded model envelopes. This process requires very high computational power. Built-in software support for generating and exporting graphical outputs would improve the usability of these analyses.

Future research should aim to incorporate additional stressors such as heatwave frequency, vapor pressure deficit, and soil salinity into soybean modeling studies. Future research could integrate remote-sensing-based calibration, evaluate alternative irrigation strategies, and explore genotype-specific adaptation pathways may further improve predictive performance. Such improvements may contribute to the development of more resilient soybean production systems and support long-term sustainable water management in semi-arid agricultural regions.

## Data Availability

The original contributions presented in the study are included in the article/supplementary material. Further inquiries can be directed to the corresponding author.
